# Editorial: Precision medicine in neurocritical care

**DOI:** 10.3389/fneur.2026.1949303

**Published:** 2026-08-13

**Authors:** Cina Sasannejad, Hitoshi Fukuda, Michael L. James

**Affiliations:** 1 Department of Neurology, Duke University, Durham, NC, United States; 2 Department of Neurosurgery, Kochi Medical School Hospital, Kochi University, Kochi, Japan; 3 Departments of Anesthesiology, Neurology, and Neurosurgery, Duke Clinical Research Institute, Duke University, Durham, NC, United States

**Keywords:** bioinformatics, biomarkers, machine learning, neurocritical care, neuroprognostication, precision medicine

## Introduction

The complexity, urgency, severity of illness, and pace of technological advancement in neurocritical care necessitate thoughtful and proactive approaches to navigating scenarios and questions in which guidelines can only guide management so far. A 2018 editorial identified endovascular thrombectomy for acute ischemic stroke as an early example of successful implementation of precision medicine in a neurocritically ill patient population, highlighting the use of advanced neuroimaging to identify individual candidates most likely to benefit from the procedure (1). More recently, advanced neuromonitoring approaches to determine and monitor optimal cerebral perfusion pressure (CPP_opt_) over moving 4-h windows have introduced individualized approaches to caring for patients with traumatic brain injury (TBI) (2) and subarachnoid hemorrhage (SAH) (3), culminating in the first randomized controlled trial of cerebral autoregulation-guided management in TBI (4). The manuscripts in this Research Topic encompass a wide variety of neurocritical care conditions, including ischemic stroke, intracerebral hemorrhage (ICH), SAH, TBI, and sepsis, and reflect multimodal approaches to identifying individual patients at risk for clinical deterioration: blood biomarkers, machine learning, and imaging, among others. The Research Topic also explores ways in which monitoring techniques, machine learning, and attention to pharmacological variability can guide individualized patient care in the intensive care unit.

## Machine learning approaches to data

He et al. describe validity of an interpretable machine learning model for prediction of venous thromboembolism (VTE) in patients with intracerebral hemorrhage (ICH). VTE is a disproportionately common complication of ICH compared to ischemic stroke, due to post-hemorrhage systemic hypercoagulopathy (5). Once VTE occurs, the threat of ICH expansion after initiating anticoagulation complicates ICU management. Applying machine learning to predictive modeling in the neurocritical care setting has precedent in aneurysmal subarachnoid hemorrhage and offers benefits in interaction detection, feature contribution, and collinearity minimization vs. traditional statistical modeling, and is set to become an increasingly used tool in the future of neurocritical care (6).

Babel et al. extend machine learning into procedural timing for tracheostomy and percutaneous endoscopic gastrostomy (PEG) tube placement, which patients in the Neuro ICU frequently require. The authors used reinforcement learning to generate individualized timing recommendations, finding that although early interventions improve outcomes overall, delaying these procedures can reduce mortality for select patients.

Finally, Katsuki et al. apply cluster analysis to profiling patients with chronic migraine according to clinical characteristics. Chronic migraine may initially seem to be less relevant to neurocritical care; however, phenotyping and clinical classification are crucial elements of making sense of patient data, and such an approach can be applied in the acute setting in the future to better characterize headache symptoms from hemorrhage expansion or worsening cerebral edema in order to identify patients at risk for clinical deterioration.

## Imaging and biomarker-based predictive modeling

Precision medicine for systemic complications of aneurysmal SAH and other neurocritical care conditions can enhance early detection of patients at risk for adverse outcomes. The predictive utility of biomarkers associated with nosocomial infection, electrolyte disorders, and cardiopulmonary complications for adverse functional outcomes has been investigated in SAH (7). Identifying cost-effective biomarkers available in routine laboratory workups can expand the accessibility of precision medicine approaches and provide practical utility in minimizing time awaiting send-out laboratory studies. Tang et al. found the ratio of uric acid to albumin (UAR) to be a useful predictor of postoperative cardiovascular events and pneumonia in patients with SAH undergoing aneurysm repair. Similarly, Sun D. et al. found moderate predictive performance in a higher serum creatinine-to-albumin ratio as a marker for increased mortality from 7 days to 1 year in 2,819 critically ill stroke patients. Interestingly, in developing a prediction model for sepsis in ICU patients with moderate to severe stroke, Sun H. et al. investigated a number of inflammatory biomarkers, including TNF-α, MMP-9, and S-100β, but ultimately found that only IL-10 survived alongside more readily available information including hyperlipidemia, serum creatinine, and NIHSS score. Continuing the theme of nomogram development to predict infection risk, Yang et al. analyzed 612 patients undergoing intracranial aneurysm surgery, identifying pneumonia, external ventricular drainage, tracheostomy, procalcitonin, C-reactive protein, and albumin as independent predictors of postoperative intracranial infection, although external validation will still be needed.

The manuscripts in this Research Topic demonstrate attempts to synergize the multiple types and sources of clinical information available in the ICU. Li et al. integrate lab values with clinical and imaging data to develop and externally validate a nomogram to predict poor 90-day functional outcomes in patients with ICH, incorporating uric acid, blood glucose, and hemoglobin along with Glasgow Coma Scale score and hematoma location. Another form of imaging gains predictive utility in predicting thromboembolic complications in Ma et al., in which the authors found that spontaneous echo contrast, age, and albumin predicted venous thromboembolism in 69 ICU patients with severe intracerebral hemorrhage. This study is especially interesting because VTE prevention after ICH remains difficult in practice, and spontaneous echo contrast may offer a useful early signal for more individualized thromboprophylaxis despite the small single-center cohort. Finally, Song et al. show in a MIMIC-IV cohort of 1,643 patients that higher heart rate in the initial 24 h of ICU hospitalization independently predicts 28-day mortality in patients with stroke without atrial fibrillation. While the retrospective design imparts some caution in interpreting these findings, this study highlights a practical application of a simple bedside variable to identify higher-risk stroke patients earlier.

The risk prediction models in our Research Topic are uniquely juxtaposed against three systematic reviews of risk prediction modeling. Two manuscripts systematically review prediction models of sepsis-associated encephalopathy (SAE). Shen et al. and Zhou et al. both identified high risk of bias, methodological flaws, and insufficient external validation as factors limiting routine clinical use of SAE prediction modeling beyond exploratory applications. Similarly, Zhang et al.'s evaluation of 12 studies comprising over 45,000 patients found that although many models showed strong discrimination, all had high risk of bias on PROBAST assessment. These systematic reviews collectively provide an important reminder of the limitations of the current state of prognostic tools in neurocritical care, encouraging standardizing common data elements and conducting prospective, multicenter studies guided by PROBAST and TRIPOD guidelines.

## Monitoring and individual variability

Rosinhas et al. shift our attention from prediction to data acquisition and monitoring. In 19 critically ill adults, the authors found good agreement between a wearable device and standard ICU monitoring for heart rate and pulse, but weaker performance for oxygen saturation, temperature, and especially blood pressure, suggesting promise for less invasive monitoring but not readiness to replace conventional systems. Wang et al. present an ambitious multimodal monitoring strategy in 200 critically ill patients with SAE. By combining microcirculatory, EEG, and biomarker data, the authors achieved an integrated model AUC of 0.92, supporting the idea that complex brain dysfunction in sepsis may be better captured through combined physiologic monitoring, though implementation may be limited by resource requirements.

As prediction and monitoring must be directed toward individual physiology to fully implement precision medicine in neurocritical care, Mahmoud et al. highlight the importance of recognizing pharmacological variability among, and within, individuals. In characterizing pharmacological variability, its contributing factors, and consequences including therapy failure or adverse drug effects, the authors illustrate the complex physiological events and differences that can guide individualized approaches to pharmacotherapy in the ICU.

## Future directions

In a review of stroke-based precision medicine in neurocritical care, Petersen et al. identified several key components requiring advancement: harnessing and processing data, developing biomarkers and clinical phenotypes, and applying physiology-based monitoring to personalized treatment targets (8). The manuscripts in this Research Topic focus predominantly on using data to identify patients at risk for complications and adverse outcomes, incorporating both machine learning and predictive models of biomarkers. As applications of precision medicine approaches in neurocritical care continue to grow, this focus will increasingly shift from prediction and monitoring to building evidence for targeted treatment strategies that can inform future guidelines. We thank the authors and reviewers for their work.
